# NPR-9, a Galanin-Like G-Protein Coupled Receptor, and GLR-1 Regulate Interneuronal Circuitry Underlying Multisensory Integration of Environmental Cues in *Caenorhabditis elegans*

**DOI:** 10.1371/journal.pgen.1006050

**Published:** 2016-05-25

**Authors:** Jason C. Campbell, Lauren F. Polan-Couillard, Ian D. Chin-Sang, William G. Bendena

**Affiliations:** 1 Department of Biology, Queen’s University, Kingston, Ontario, Canada; 2 Centre for Neuroscience, Queen’s University, Kingston, Ontario, Canada; Katholieke Universiteit Leuven, BELGIUM

## Abstract

*C*. *elegans* inhabit environments that require detection of diverse stimuli to modulate locomotion in order to avoid unfavourable conditions. In a mammalian context, a failure to appropriately integrate environmental signals can lead to Parkinson’s, Alzheimer’s, and epilepsy. Provided that the circuitry underlying mammalian sensory integration can be prohibitively complex, we analyzed nematode behavioral responses in differing environmental contexts to evaluate the regulation of context dependent circuit reconfiguration and sensorimotor control. Our work has added to the complexity of a known parallel circuit, mediated by interneurons AVA and AIB, that integrates sensory cues and is responsible for the initiation of backwards locomotion. Our analysis of the galanin-like G-protein coupled receptor NPR-9 in *C*. *elegans* revealed that upregulation of galanin signaling impedes the integration of sensory evoked neuronal signals. Although the expression pattern of *npr-9* is limited to AIB, upregulation of the receptor appears to impede AIB and AVA circuits to broadly prevent backwards locomotion, i.e. reversals, suggesting that these two pathways functionally interact. Galanin signaling similarly plays a broadly inhibitory role in mammalian models. Moreover, our identification of a mutant, which rarely initiates backwards movement, allowed us to interrogate locomotory mechanisms underlying chemotaxis. In support of the pirouette model of chemotaxis, organisms that did not exhibit reversal behavior were unable to navigate towards an attractant peak. We also assessed ionotropic glutamate receptor GLR-1 cell-specifically within AIB and determined that GLR-1 fine-tunes AIB activity to modify locomotion following reversal events. Our research highlights that signal integration underlying the initiation and fine-tuning of backwards locomotion is AIB and NPR-9 dependent, and has demonstrated the suitability of *C*. *elegans* for analysis of multisensory integration and sensorimotor control.

## Author Summary

Multiple environmental cues are sensed by an organism in order to coordinate behavioral responses. Consequently, organisms must be able to simultaneously detect and integrate multiple external stimuli in order to appropriately modify their behavior. Identifying the unique circuits mediating the response to individual stimuli and points of overlap is essential to understanding how multiple signals can be integrated for a coordinated behavioral response. In order to analyze individual circuits, we have used the model organism *C*. *elegans*. We have identified that a *C*. *elegans* neuropeptide receptor (NPR-9) and a glutamate receptor (GLR-1) function in a single interneuron to play a broad regulatory role in multiple neural circuits. Our research has identified that interneuron AIB is involved in the integration of signals from numerous sensory neurons. Moreover, regulation of AIB via a neuropeptide receptor (NPR-9) and a glutamate receptor (GLR-1) coordinates AIB activity in the context of multisensory integration. Long-range chemotaxis behavior, in which an organism alters locomotory patterns based on odorant sensation, is also regulated by NPR-9. Our analysis indicates that reversals, and thus the pirouette model, are sufficient for chemotaxis.

## Introduction

Neuronal circuits integrate various environmental stimuli to alter behavioral phenotypes. Characterization of the mechanisms underlying multisensory integration is essential to understanding how organisms perceive their environment. Disruptions to neural signaling pathways have been linked to a variety of disorders including epilepsy and schizophrenia [1,2]. Although mammalian models have provided useful insights, the complexity of the vertebrate nervous system coupled with limitations in cell specific analysis have impeded the characterization of neural circuits. Alternatively, *C*. *elegans* has proven to be a model organism for neuroscience due its established connectome, relatively simple nervous system of 302 neurons, and an ability to respond to an array of environmental conditions [3–7]. Individual neuronal pathways have been identified for distinct nematode behaviors, yet our understanding of how multiple environmental cues are integrated and can reconfigure signaling pathways is limited. For instance, nematodes respond slower to volatile odorants when a food source is not available [6]. Evaluation of behavior in the presence of multiple environmental cues provides a means to analyze how distinct cues can modify neuronal circuits. Indeed, mammalian studies have identified that seemingly unrelated cues (auditory and visual) have pronounced effects on the perception of the observer [8].

Environmental stimuli alter nematode locomotory patterns, e.g. backwards and forwards locomotion, via unique pathway [6,9]. For instance, mechanical and odorant stimuli are detected by the two ASH sensory neurons which modulate the activity of interneurons AIB and AVA to promote the initiation of backwards locomotion, i.e. a reversal [6,9]. Despite the shared neuronal contributions, the signaling pathways are differentially modulated by alternative environmental cues that are simultaneously integrated. More specifically, the presence of food alters neuronal activity and responsiveness to an odorant, while signaling dynamics underlying mechanical stimulation remain unaffected [6,10]. Understanding the mechanisms, i.e. neurons and signaling molecules, that allow for differential modulation of sensory pathways is essential to the field of multisensory integration.

Interneurons AVA and AIB function in parallel to trigger the initiation of backwards locomotion upon their activation [9]. However, AIB expresses serotonin, glutamate, and neuropeptide receptors, while AVA is only known to integrate glutamatergic signaling [11–13]. Mutants that lack a functional vesicular glutamate transporter, encoded by *eat-4*, are largely defective for the initiation of a reversal in response to environmental stimuli [14]. However, multiple neurotransmitters and neuropeptides are released to regulate circuit activity [15,16]. Consequently, AIB has the unique potential to integrate diverse, context-dependent signals from sensory neurons. AIB is also densely connected to the interneuronal circuitry highlighting its capacity for a broad role in the regulation of downstream or parallel pathways (Table 1) [3]. AIB has been identified as a hub for the integration of signals derived from sensory neurons ASH, AWC and ASE in the regulation of octanol responses on and off food [17].

**Table 1:** Post-synaptic connectivity of interneuron AVA and AIB to sensory neurons.

Neuron	Sensory connectivity
AVA	FLP, ASH, AWC, BAG
AIB	FLP, ASK, ASI, ASH, ASG, ASE, AWC, AWB, BAG

Within this study, we have expanded the range of behaviors analyzed in order to determine if AIB serves as a broad integration hub or is specific to octanol. Since glutamate is an essential signaling molecule of many behaviors and has been shown to regulate AIB activity in response to environmental cues, we evaluated *glr-1* cell specifically across a range of behaviors [9,12,17]. Interactions between galanin and glutamate signaling pathways have been highlighted in mammalian models [18]. Hence, we also investigated a galanin-like G-Protein Coupled Receptor (GPCR), namely NeuroPeptide Receptor-9 (NPR-9). NPR-9 is expressed exclusively in AIB and modulates spontaneous locomotion. Loss of function alleles of *npr-9* produce an increase in “dwelling” behavior characterized by a lack of forward locomotion, while over-expression of *npr-9* increases forward locomotion [13].

Loss of function of *npr-9* alters spontaneous reversal frequencies on and off food, while also producing abnormal off food octanol responses. *glr-1* knock-down (KD) specifically in AIB only interferes with off food octanol responses, however *glr-1* (KD) in an *npr-9(LF)* background alters spontaneous locomotion frequencies. Over-expression of *npr-9* abolishes the initiation of a reversal in response to nose touch, octanol, copper, or during spontaneous locomotion. Ablation of either AVA or AIB reduces spontaneous reversal frequency by ~50%. Ablation of both interneurons nearly abolishes reversals suggesting that AVA and AIB function in parallel pathways to initiate a reversal [9]. Over-expression of *npr-9* mimics the dual ablation of AVA and AIB. Provided that *npr-9* is only expressed in AIB, the *npr-9(GF)* phenotype suggests that NPR-9 can regulate both pathways.

Moreover, identification of a mutant that rarely reverses has also allowed us to interrogate the behavioral mechanisms underlying long-range chemotaxis. The weathervane model of chemotaxis purports that nematode turning behavior mediates chemotaxis, while the pirouette model suggests that reversals are responsible [19–21]. Mutants exhibiting abnormal turning behavior exhibit normal chemotaxis responses, which has cast doubt on the weathervane model [22]. Here we report that the pirouette model is the primary mode of nematode chemotaxis. Collectively, our research indicates that NPR-9 is a key regulator of AIB, which serves to integrate signals from multiple sensory neurons and coordinate the interneuronal circuitry to control locomotion.

## Results

### The presence of food modifies *npr-9(LF)* locomotory patterns

Food availability serves as an environmental cue to modify spontaneous and evoked behavioral patterns. Nematodes move slower in the presence of food and exhibit less reversals compared to organisms moved to environments absent of food [23,24]. These behavioral changes are associated with food availability and are largely regulated by serotonin signaling, i.e. serotonin signaling is up-regulated while organisms are on food [23]. Indeed, the mutant *tph-1*, which is unable to biosynthesize serotonin, exhibits abnormal locomotory patterns during the transition from a well-fed to starved state [24, 25]. Inhibitory serotonin receptor MOD-1 is expressed in AIB and plays a role in the food-based serotonergic regulation of locomotion [11,17, 26]. AIB has been implicated in mediating the transition from well-fed to starved behavioral states [24,27].

**Fig 1:**
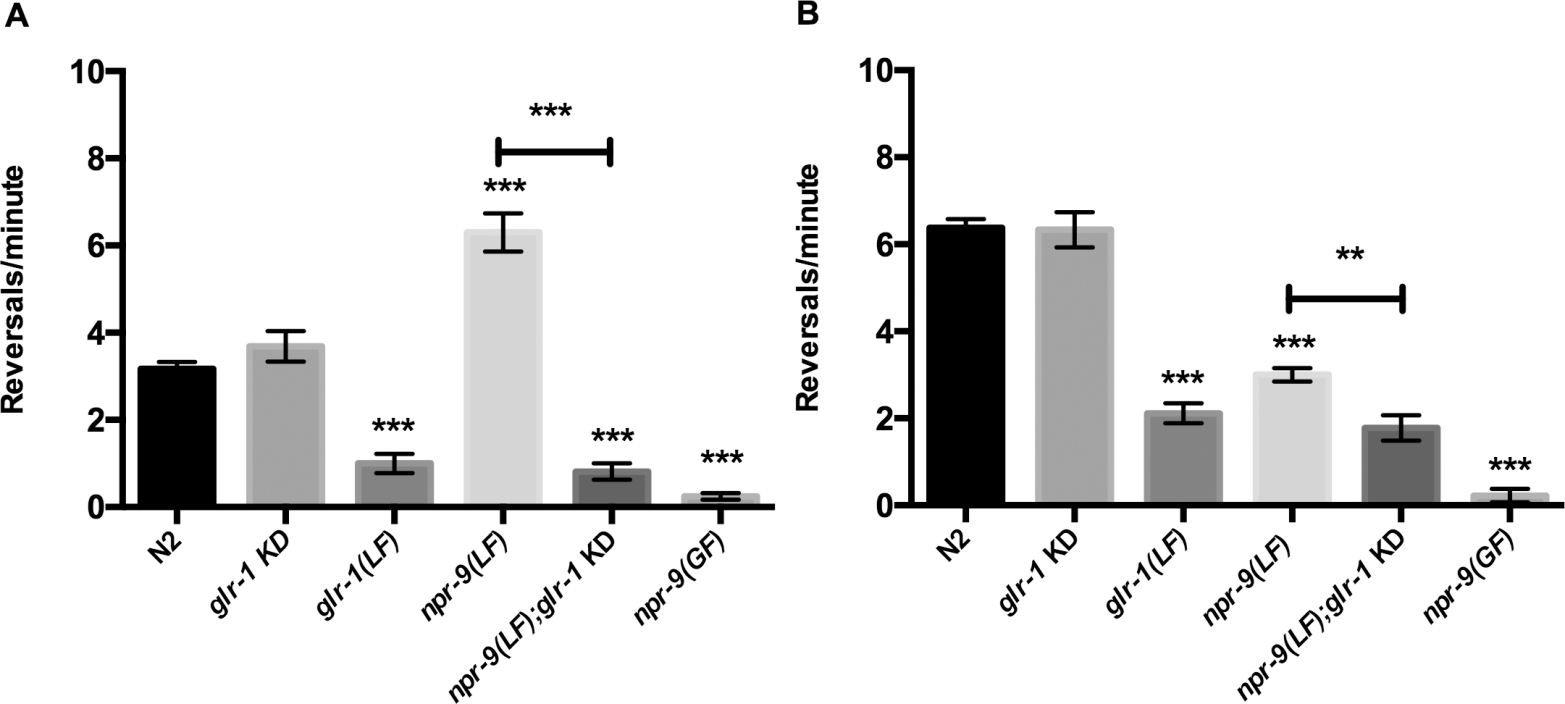
On and off food reversal frequencies are regulated by NPR-9 and GLR-1 in the AIB. (A) Reversal phenotypes over the course of 3 minutes on food in wildtype (N2), *glr-1* knockdown in the AIB (*glr-1* KD), *npr-9(LF)*, *npr-9(LF);glr-1* KD, *glr-1(LF)*, and *npr-9(GF)*. (B) Off food reversal frequencies of N2, *glr-1* KD, *npr-9(LF)*, *npr-9(LF);glr-1* KD, and *npr-9(GF)*. Data are presented as mean +/- standard error (SE) with at least 5 animals assayed in three independent experiments analyzed via an unpaired two-tailed *t* test with Welsch’s correction. *** p < .001, ** p< .01, significantly different from wild-type animals under identical conditions. Asterisks above error bars indicate significantly different between the two strains.

Manipulations to NPR-9 dependent signaling alter general roaming behavior, however the effect on reversal frequency remains uncharacterized. In order to isolate the specific locomotory defect of *npr-9* mutants, we evaluated reversal frequency. Behavioral analysis with and without food has aided in the characterization of context dependent circuit reconfiguration. Wildtype *C*. *elegans* exhibit spontaneous reversals on food 3 times per minute and 6 times per minute off food [28,29] (Fig 1A). In contrast, *npr-9(LF)* animals exhibit a decreased reversal frequency off food and an increased reversal frequency on food (Fig 1A and 1B). The abnormal reversal frequency of *npr-9(LF)* mutants indicates that the neuropeptide receptor coordinates circuit activity yet promotes differing locomotory patterns based upon environmental cues.

### Loss of GLR-1 in AIB does not alter reversal frequency

Decreases in glutamatergic signaling, as observed in mutants defective for glutamatergic transmission (e.g. loss of glutamate receptor GLR-1), decreases reversal frequency both on and off food [29,30]. Interneuron specific expression of *glr-1* suppresses locomotory defects in *glr-1* mutants indicating that glutamate regulates interneuron activity to control locomotion [29,30]. The similar on and off food phenotypes suggest that glutamatergic regulation is independent of nutritional state. However, a metabotropic glutamate receptor within AIB is essential in the regulation of starvation responses [27]. Consequently, the role of glutamate in regards to contextual regulation of locomotion remains unclear. In order to evaluate the role of GLR-1 cell specifically, we knocked-down expression of *glr-1* within AIB. Organisms that lacked AIB *glr-1* expression exhibited no change in reversal frequency. Provided that a parallel glutamatergic pathway is mediated by AVA and AIB, the lack of *glr-1* expression is likely compensated by GLR-1 activity in AVA. Interestingly, AIB or AVA ablation reduces reversal frequency by ~50%, yet the lack of a stimulatory glutamate receptor in AIB has no effect [9]. This suggests that alternative receptors are able to maintain endogenous AIB activation patterns.

### NPR-9 over-expression inhibits spontaneous reversals

Ablation of either AIB or AVA reduces on food reversal frequency by approximately 50%, but does not abolish reversals entirely. Collective ablation of these interneurons reduces reversal frequency by ~80% indicating that they promote reversals independently. AIB activation leads to inhibition of motor neuron RIM to trigger the initiation of backwards locomotion [9]. Organisms with ablated AIBs also display decreased reversal frequencies off food suggesting that AIB activation promotes reversals in either context [24].

Over-expression of *npr-9* led to more than a 90% reduction in reversal frequency both on and off food (Fig 1A and 1B). Under standard locomotion assay conditions, AIB ablation or AIB-expression of cell death gene *egl-1* reduces reversal frequency by roughly 50% [9,17,24]. Consequently, the severe lack of reversals exhibited by *npr-9(GF)* organisms suggests that NPR-9 regulates AVA in a cell non-autonomous manner (Fig 1A and 1B). Although AVA and AIB work in parallel to initiate a reversal, regulatory interactions have been identified between the two pathways [3,24]. Regulation of AVA is largely mediated by glutamatergic signaling while AIB activity is dictated by a number of signaling molecules suggesting that AIB and AVA circuits are not redundant [11–13,30]. Cross-talk between AVA and AIB mediated circuits would ensure that AIB signal integration is incorporated to fine-tune locomotion. Such cross-talk could include synaptically transmitted glutamate or peptides that are expressed in interneurons.

### Glutamate and NPR-9 regulate omega turns

Omega turns are a component of *C*. *elegans* locomotion in which the nematode exhibits a 135° change in direction resembling an omega (Ω) symbol. These turns are characteristically observed after off-food reversals of three or more head swings, termed “long reversals” [24]. Null *glr-1* mutants exhibit a decrease in the number of omega turns per minute [31]. Similar to the nose touch pathway, glutamate is likely the major coordinator of omega turns.

Since omega turns occur at the end of a reversal, the regulation of omega turns is likely independent from the signaling pathway underlying the initiation of backwards locomotion. Analysis of omega turn frequency thus allows for the interrogation of neuronal fine-tuning independent of reversal initiation. Organisms with ablated AIB neurons exhibit a reduction in omega turns indicating that AIB promotes omega turn behavior, while AVA ablations do not alter omega turn patterns [24]. Prior analyses of omega turns have characterized the behavior by measuring either the frequency of omega turns or the absolute number [24,31]. For instance, an organism that exhibited 6 reversals and 3 omega turns in three minutes would be calculated as 50% with our research parameters, while absolute measurements would present the data as 1 omega turn/minute. However, an organism that reversed 30 times and exhibited 3 omega turns would still be represented as 1 omega turn per minute, while we would present that as 10%. We calculate omega turn frequency relative to the total number of reversals in order to determine how frequently an omega turn occurs after a reversal as opposed to counting omega events without consideration for reversal frequency. Considering omega turns are coupled to reversals, the number of omega turns per minute is dependent on reversal frequency. Thus, measuring the absolute number of omega turns simultaneously analyzes the initiation and termination of reversals, which are distinctly regulated [17]. In order to solely interrogate the circuitry regulating omega turns, we have evaluated *glr-1* KD, *glr-1* and *npr-9* mutants for omega turn frequency.

**Fig 2:**
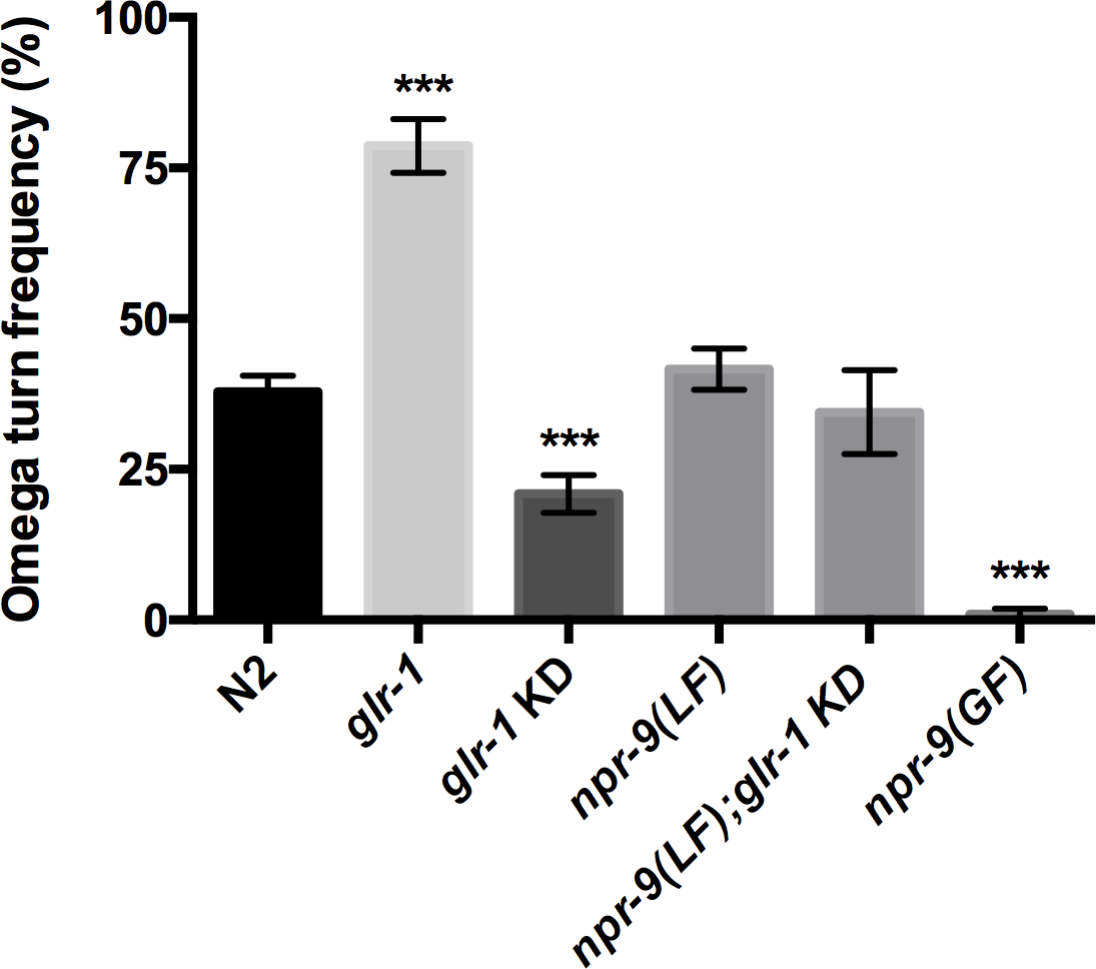
Glutamate is a key regulator of omega turns. N2, *glr-1(LF)*, *glr-1* KD, *npr-9(LF)*, *npr-9(LF);glr-1* KD, and *npr-9(GF)* were tested for the frequency of reversals that terminate with an omega turn during off food conditions Data are presented as mean +/- standard error (SE) with at least 5 animals assayed in three independent experiments analyzed via an unpaired two-tailed *t* test with Welsch’s correction. *** p < .001, significantly different from wild-type animals under identical conditions.

Wildtype organisms perform an omega turn after roughly 40% of reversals off food (Fig 2). *glr-1* null organisms exhibited an increased omega frequency (Fig 2). To the contrary, loss of *glr-1* within the AIB produced a deceased omega turn frequency (Fig 2). Consequently, GLR-1 function within AIB serves to promote omega turns while alternative neurons inhibit omega turns via GLR-1.

Despite a role in regulating off-food spontaneous reversals, *npr-9* null animals exhibited no significant change in omega turns (Fig 2). Surprisingly, the omega turn frequency of *npr-9(LF);glr-1* KD did not differ from N2 (Fig 2). This could suggest that AIB regulation of omega turns via GLR-1 is dependent on functional NPR-9 signaling. Since omega turns occur after the initiation of reversals, termination events, e.g. omega turns, are likely dependent on the upstream signaling dynamics.

Over-expression *of npr-9* decreased omega turn frequency likely due to the absence of reversals (Fig 1). We suspect that *npr-9(GF)* exhibits infrequent omega turns due to inhibition of the upstream reversal initiation. Collectively, the data indicates that NPR-9 is essential for the initiation of a reversal, while GLR-1 fine-tunes the termination of a reversal via omega turns.

### NPR-9 and GLR-1 play a role in neuronal signaling underlying nose touch responses

The initiation of backwards locomotion following mechanostimulation to the nematode nose is known as the “nose touch response”. In contrast to the signaling pathway underlying spontaneous locomotion, serotonin does not regulate the nose touch response [32]. Defective nose touch responses amongst glutamate receptor mutants (*glr-1* and *glr-2)* indicate that glutamate is the primary signaling molecule regulating the nose touch circuitry [33]. The initiation of backwards locomotion evoked from nose touch is dependent on the AVA/AIB parallel pathway that governs spontaneous locomotion reversal frequency. GLR-1 activity in either AVA or AIB is sufficient to restore the nose touch response in a *glr-1* mutant background [9].

**Fig 3:**
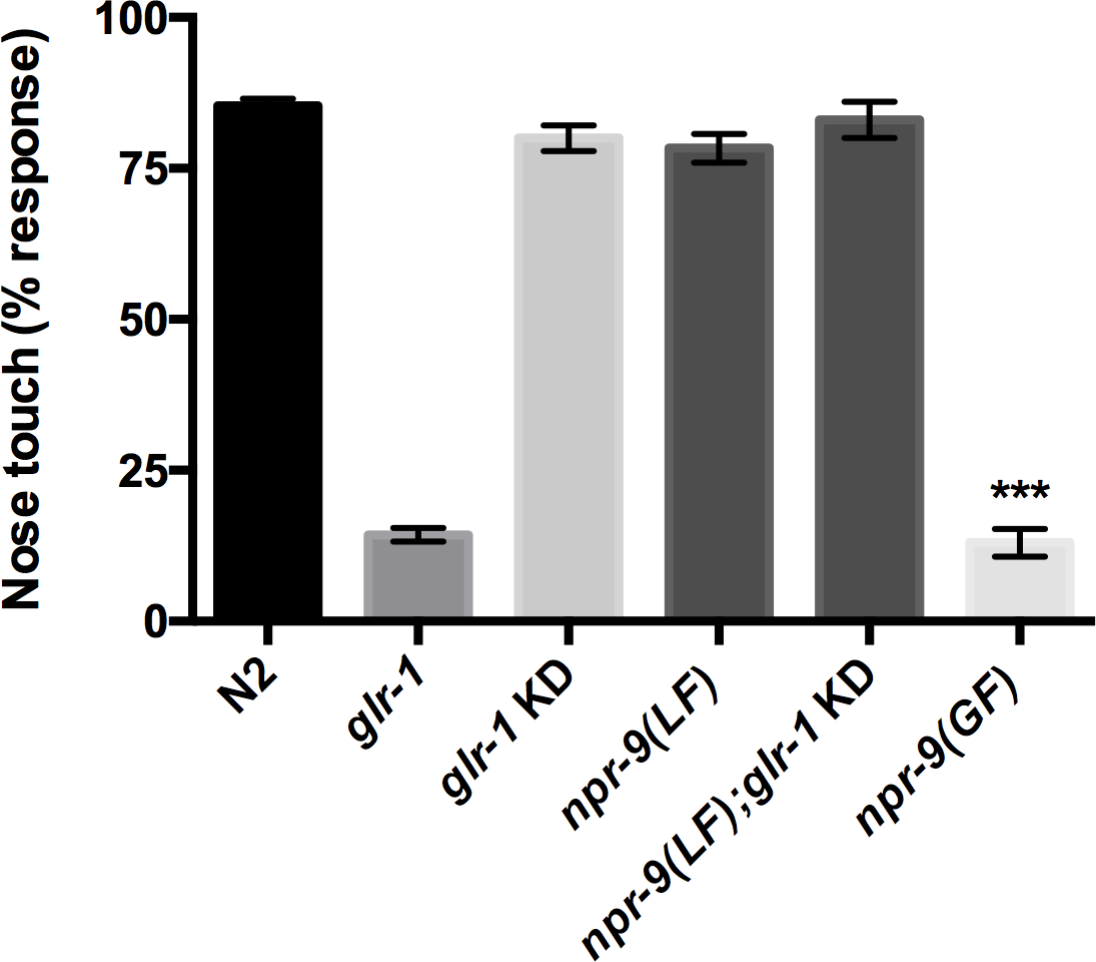
Over-expression of *npr-9* inhibits nose touch responses. Wildtype, *glr-1(LF)*, *glr-1* KD, *npr-9(LF)*, *npr-9(LF);glr-1 KD*, and *npr-9(GF)* were evaluated for ability to respond to nose touch stimulation. Data are presented as mean +/- standard error (SE) with at least 10 animals assayed in three independent experiments analyzed via an unpaired two-tailed *t* test with Welsch’s correction. *** p< .001, significantly different from wild-type animals under identical conditions.

Although AVA and AIB interneurons regulate reversal patterns, similar to spontaneous locomotion, the absence of a role for serotonin suggests that the circuit is differentially modulated. We evaluated nose touch responses of *npr-9* and *glr-1* KD mutants to delineate signaling differences between spontaneous locomotion and reversals provoked by nose touch. In agreement with the parallel pathway, *glr-1* KD in the AIB exhibited no defect in nose touch responses (Fig 3). Despite the reduction in spontaneous reversal frequency, *npr-9(LF)* and *npr-9(LF);glr-1* KD organisms exhibited wildtype nose touch responses (Figs 1A, 1B and 3). To the contrary, AIB ablation results in a reduced, although not abolished, nose touch response which suggests that abnormal AIB regulation should affect signal dynamics [3]. However, *npr-9(GF)* resembled the *glr-1* null mutant and rarely responded to nose touch stimulation (Fig 3). Despite the distinct pathways underlying spontaneous and nose touch reversals, *npr-9(GF)* fails to reverse in either context which suggests that signals downstream of NPR-9 broadly inhibit multiple circuits.

### GLR-1 and NPR-9 play a unique role in the octanol response

Octanol is a volatile odorant that stimulates backwards locomotion. In the presence of food, organisms respond to dilute (30%) octanol within 3–5 seconds. Without food in the environment, nematodes initiate backwards locomotion within 8–10 seconds [34]. Application of exogenous serotonin, in the absence of a bacterial lawn, can restore on food responses [34]. A complex network of neuropeptides and neurotransmitters regulate dilute octanol responses on and off food [15,16].

Sensory neuron AWC basally stimulates AIB via GLR-1, while ASE derived glutamate acutely inhibits AIB activity AVR-14. Moreover, MOD-1 functions to basally inhibit AIB activity while organisms are on food [17]. Collectively these results indicate that AIB is an integration hub for signals derived from differing sensory neurons that act antagonistically to regulate AIB activity. The integration of multiple signals allows for “fine tuning” of backwards locomotion, i.e. dictating the trajectory of movement after reversal termination.

**Fig 4:**
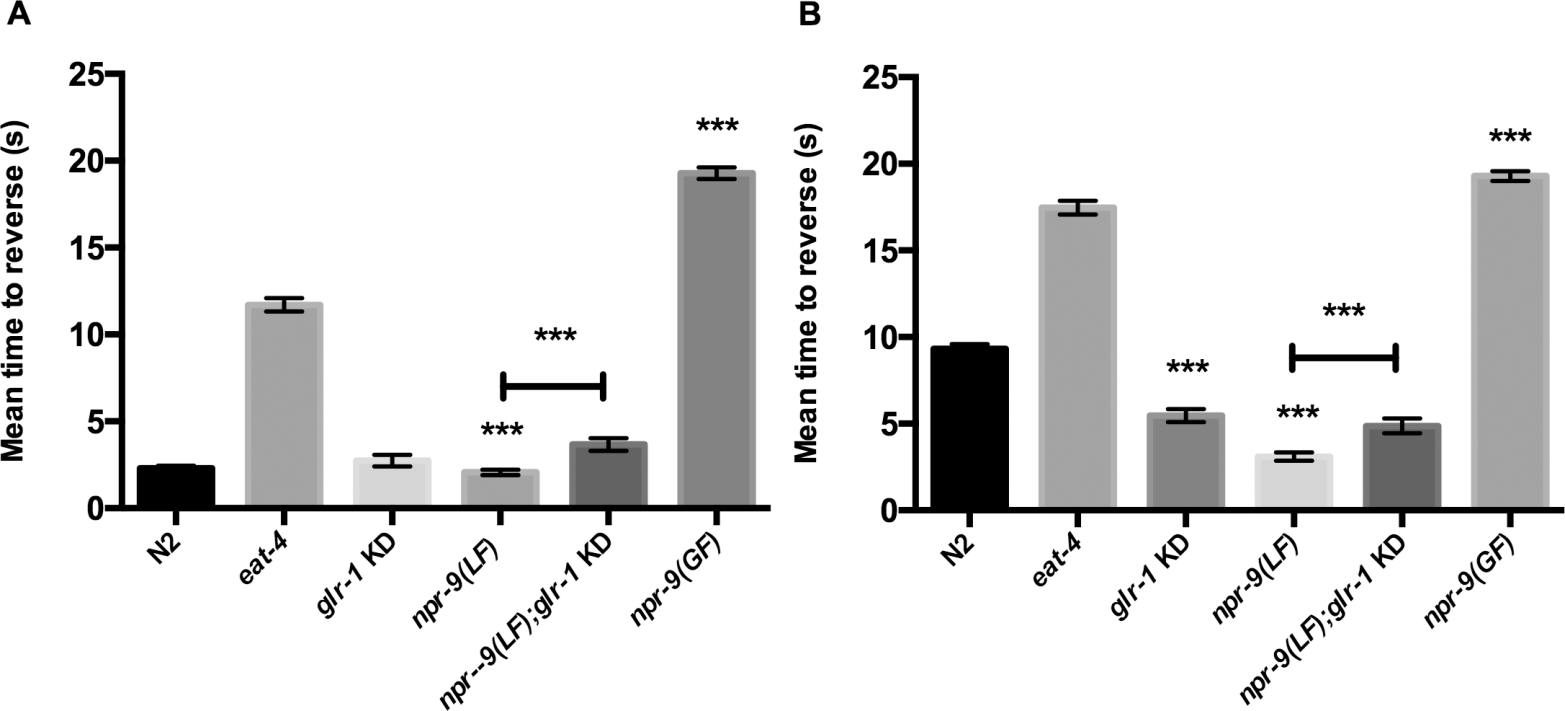
NPR-9 and GLR-1 signaling are non-essential for on food 30% octanol behavior, but play a role in off food 30% octanol responses. (A) N2, *eat-4*, *glr-1* KD, *npr-9(LF)*, *npr-9(LF);glr-1* KD, and *npr-9(GF)* were assayed for responses to 30% octanol on food. (B) The aforementioned strains were evaluated for responsiveness to 30% octanol off food. Data are presented as mean +/- standard error (SE) with at least 10 animals assayed in three independent experiments analyzed via an unpaired two-tailed *t* test with Welsch’s correction. *** p < .001, significantly different from wild-type animals under identical conditions. Asterisks above error bars indicate significantly different between the two strains.

Since *npr-9* null animals have been shown to display hyper-aversive responses to dilute octanol off food, we evaluated octanol response [15]. In agreement with previously published research, *glr-1* KD and *npr-9(LF)* organisms displayed a hyper-aversive off food response (Fig 4B). However, *npr-9(LF);glr-1* KD responded slower than *npr-9(LF)* on and off food, but did not differ from *glr-1* KD in either condition (Fig 4A and 4B). The hyper-aversive *npr-9(LF)* and *glr-1* KD octanol phenotype suggest that NPR-9 and GLR-1 inhibit the initiation of reversals during off food octanol conditions. Loss of both receptors in AIB continues to produce a hyper-aversive phenotype that resembles *glr-1* KD. This suggests that signaling downstream of NPR-9 activation is partly dependent on GLR-1 regulation of AIB activity in the octanol pathway. In agreement with an inhibitory role for NPR-9, *npr-9(GF)* mutants did not respond to 30% octanol on or off food (Fig 4A and 4B).

### Over-expression of NPR-9 does not inhibit all reversals

**Fig 5:**
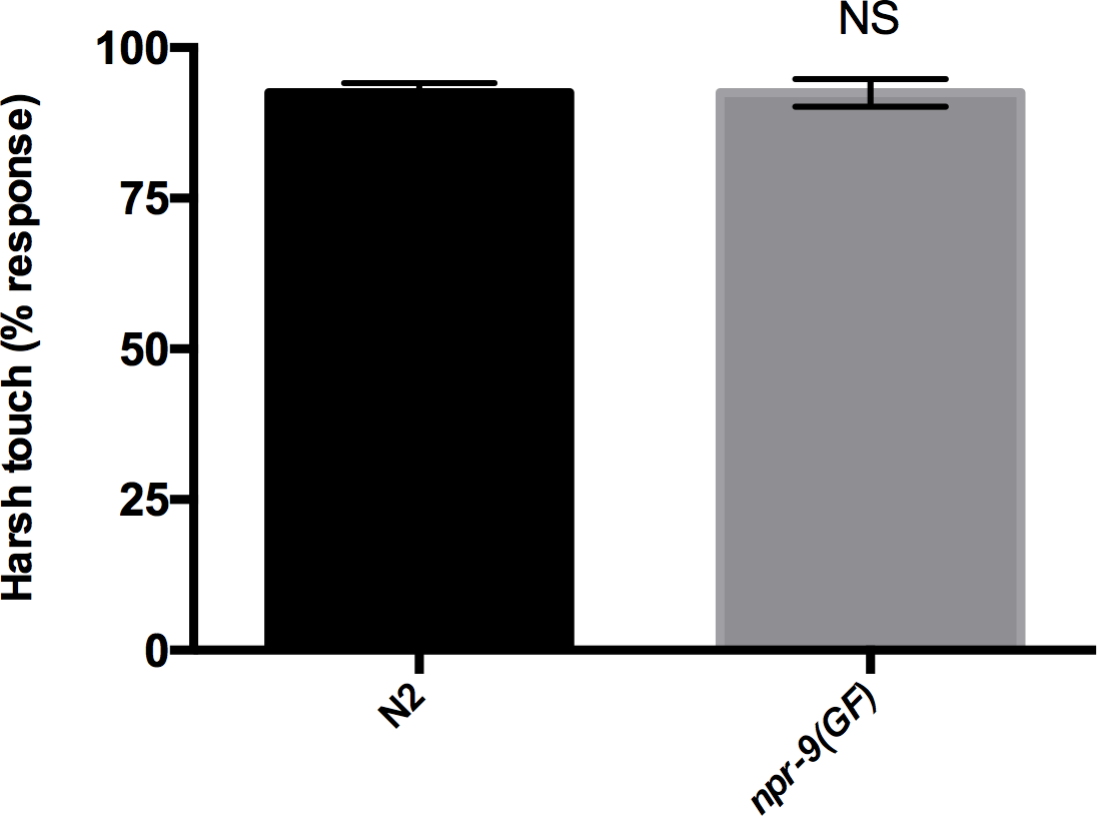
*npr-9(GF)* initiates reversals in response to harsh touch. Wildtype and *npr-9(GF)* organisms do not differ in their responses to mechanostimulation via harsh touch. Data are presented as mean +/- standard error (SE) with at least 5 animals assayed in three independent experiments analyzed via an unpaired two-tailed *t* test with Welsch’s correction. NS = non-significant.

The lack of reversals exhibited by *npr-9(GF)* could be due to the inhibition of signaling pathways that promote backward locomotion or simply due to impaired muscle contraction. AIB is pre-synaptic to AVA, which could facilitate the transmission of inhibitory signals after NPR-9 activation to inhibit reversals [3]. Thus, we sought to analyze *npr-9(GF)* for a behaviour that was not mediated by interneurons that are post-synaptic to AIB. The harsh touch response, in which prodding an organism with a wire pick anterior to the vulva induces a reversal, is primarily mediated by AVD, while an AVA ablation produces no defect [35]. AIB does not exhibit gap junctions or chemical synapses with AVD [3]. To determine if the *npr-9(GF)* defect is due to a physiological or signaling abnormality, we evaluated the mutant for the harsh touch response. Application of harsh touch to *npr-9(GF)* mutants induced reversals at wildtype levels (Fig 5). The positive harsh touch response suggests that the lack of reversals in response to differing stimuli is reflective of circuit miscoordination rather than a physiological inability to reverse. Moreover, this result reinforces the hypothesis that if NPR-9 is exclusively expressed in the AIB then it likely inhibits multiple interneurons via the transmission of signaling molecules through synaptic connections. Similar to the harsh touch response, the AVD is the primary mediator of reversals evoked from the plate tap [21,36].

### Misregulation of the interneuronal circuitry does not alter diacetyl chemotaxis

**Fig 6:**
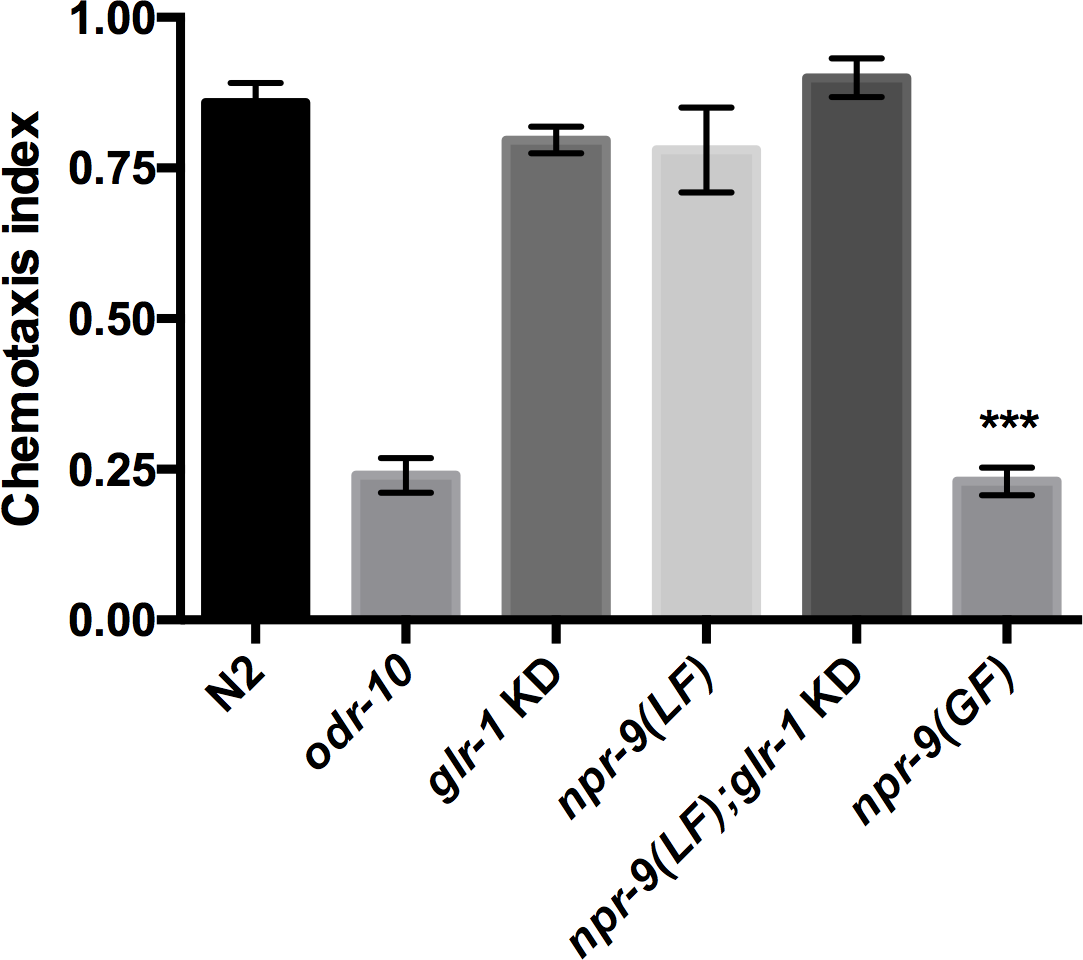
*npr-9(GF)* is not attracted to low concentrations of diacetyl. N2, *odr-10*, *glr-1* KD, *npr-9(LF)*, *npr-9(LF);glr-1* KD, and *npr-9(GF)* were assessed for chemotaxis to low concentration of diacetyl. Data are presented as mean +/- standard error (SE) with at least 50 animals assayed in three independent experiments analyzed via an unpaired two-tailed *t* test with Welsch’s correction. *** p < .001, significantly different from wild-type animals under identical conditions.

At low concentrations, diacetyl is a chemoattractant to *C*. *elegans* [37]. *odr-10* is a GPCR expressed in AWA that is essential for diacetyl chemotaxis [38]. Analysis of the interneurons that are involved in diacetyl chemotaxis has revealed a complex network of regulation. Inhibition or activation of interneurons AIB, AIY, AIZ, and AIA can alter an organism’s ability to exhibit chemotaxis to high or low concentrations of diacetyl [39]. These results indicate that complex interneuronal regulation is essential for the regulation of locomotory responses underlying general chemotaxis behavior. In order to determine the importance of circuit regulation in chemotaxis, we analyzed *npr-9(LF)* and *glr-1* KD organisms. Despite the irregular locomotory patterns, *npr-9(LF)*, *glr-1* KD, and *npr-9(LF);glr-1* KD all responded to diacetyl (Fig 6). In the context of diacetyl, it appears that loss of *npr-9* and/or *glr-1* in the AIB does not interfere with the integration of diacetyl-derived signals.

### Reversal frequency is essential for attraction to diacetyl in support of the pirouette model of chemotaxis

*C*. *elegans* chemotaxis is also mediated by locomotory behaviours; however, the behavioral mechanism underlying attraction is an issue of debate. Two theories have been proposed to explain chemotaxis: the weather vane model and the pirouette model [19–22]. The weathervane model purports that nematode attraction is mediated by gradual turns that allow a nematode to sense concentration changes and alter direction accordingly. On the other hand, the pirouette model suggests that chemotaxis is mediated by sharp changes in direction and reversals, i.e. pirouettes.

To determine the role of reversals and the pirouette model in chemotaxis, we evaluated *npr-9(GF)* for diacetyl chemotaxis (Fig 6). The lack of reversals within *npr-9(GF)* mutants translated to a defective chemotaxis response in support of the pirouette model of chemotaxis. Although *npr-9(GF)* mutants rarely reverse, they do exhibit turning behavior, thus the chemotaxis defect is most likely due to the reversal abnormality [13].

### Mechanosensory stimulation of reversals can rescue the *npr-9(GF)* diacetyl defect

**Fig 7:**
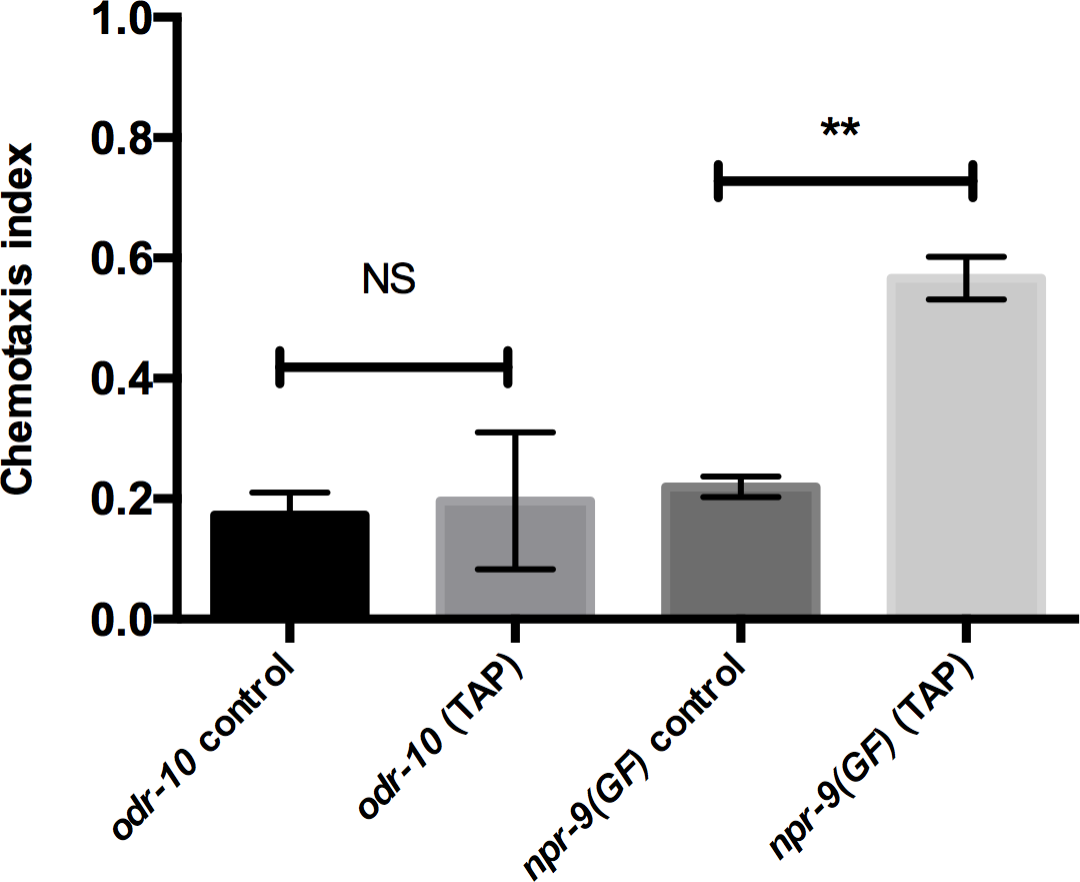
Inducing reversals with plate tap stimulation improves diacetyl responsiveness in *npr-9(GF)* organisms. *odr-10* and *npr-9(GF)* populations were evaluated for the diacetyl response, while plate taps were performed. Data are presented as mean +/- SE and analyzed with an unpaired two-tailed t test with Welsch’s correction. ** p< .01, significantly different from *odr-10* animals under identical conditions. NS = non-significant. Asterisks above error bars indicate significantly different between the two strains.

As previously mentioned, a plate tap can induce the initiation of backwards locomotion in *npr-9(GF)* mutants. To further determine the importance of reversal behavior as a mediator of chemotaxis, we incorporated a mechanosensory stimulus, i.e. a plate tap, into the diacetyl assay. If the *npr-9(GF)* diacetyl defect is derived solely from a lack of reversals, the induction of reversals via an alternative stimulus should restore diacetyl responsiveness. In support of this hypothesis, consistent administration of plate taps throughout the diacetyl assay increased chemotaxis responses in *npr-9(GF)* mutants (Fig 7). Since the diacetyl defect of *odr-10* is due to an inability to detect low concentration diacetyl, the induction of reversals should not alter the phenotype. As expected, *odr-10* mutants remain defective despite evoked reversal behavior (Fig 7).

### The initiation of reversals does not negatively affect chemotaxis

**Fig 8:**
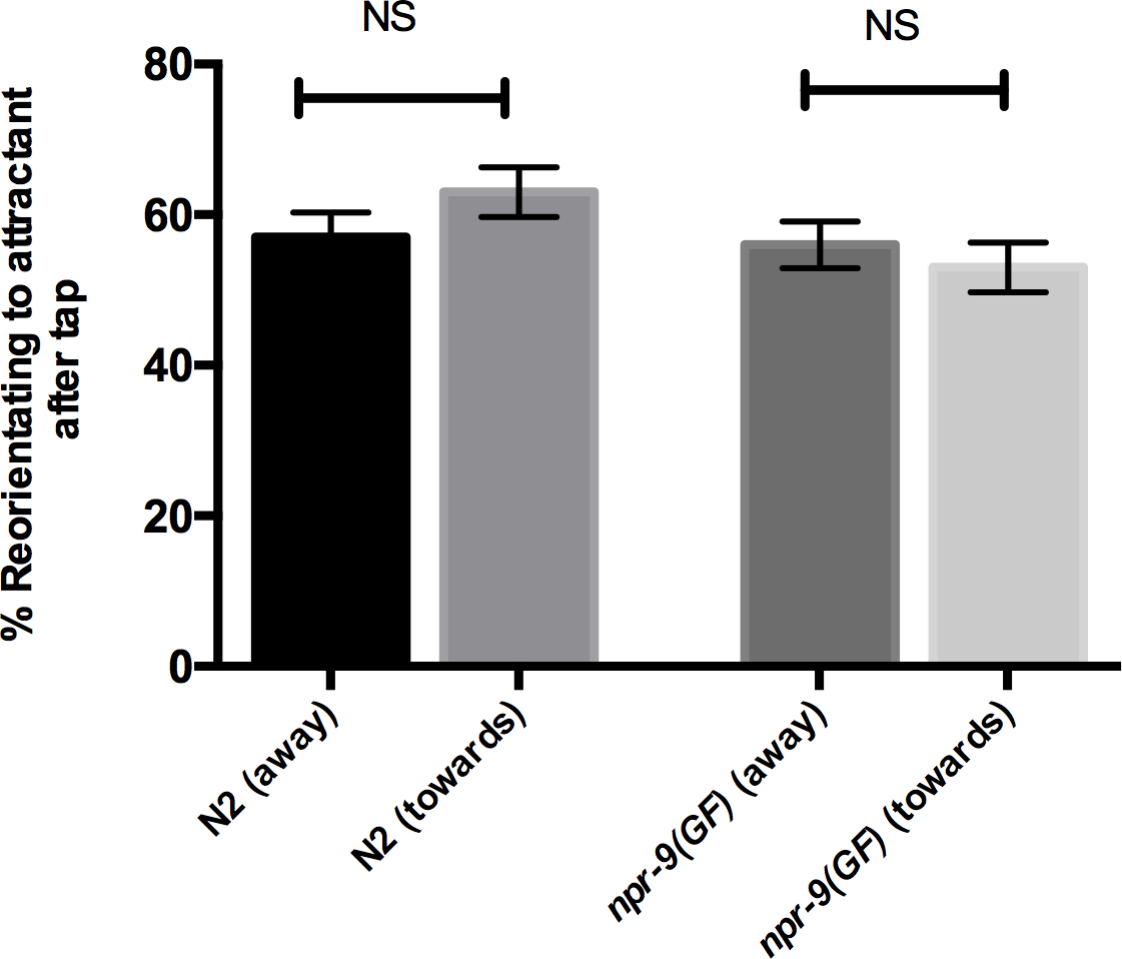
Inducing reversals with plate tap stimulation does not negatively affect chemotaxis. N2 and *npr-9(GF)* were evaluated for individual diacetyl responses, while plate taps were performed. Data are presented as mean +/- standard error (SE) with one animal assayed in 20 independent experiments analyzed via an unpaired two-tailed *t* test with Welsch’s correction NS = non-significant. Asterisks above error bars indicate significantly different between the two strains. (away) indicates that organisms were initially oriented away from the attractant peak before plate tap, while (towards) indicates that organisms were initially moving towards the attractant peak before plate tap.

Observational analyses have noted that nematodes are less likely to initiate a reversal when moving towards a higher concentration of an attractant [20]. The introduction of a plate tap during diacetyl chemotaxis assays with large sample sizes likely triggers backwards locomotion in nematodes that are moving towards and away from an attractant. Consequently, worms that are already oriented towards an attractant could be negatively affected by inducing a reversal. We introduced plate taps in assays that evaluated individual nematode responses as they moved towards and away from an attractant. N2 and *npr-9(GF)* organisms exhibited no difference in their ability to re-orient towards an attractant regardless of their initial trajectory (Fig 8). We also established that reversals increase the likelihood that organisms reorient towards an attractant; however, a reversal did not guarantee that an organism will immediately re-orient to an attractant. The behavior is likely repeated throughout the course of chemotaxis until a nematode reaches the attractant peak. Ultimately, locomotory patterns, i.e. turning and reversals, must be a component of chemotaxis as they are integral to nematode locomotion. However, in regards to the initial detection of diacetyl, reversal behavior alone is sufficient for wildtype chemotaxis.

### Upregulation of *npr-9* interferes with the integration of food cues and starvation signaling

ASH neurons also mediate copper aversion; G protein GPA-3 mediates responses to copper while ODR-3 transduces signals related to osmotic and mechanical avoidance [40–42]. The introduction of copper in the presence of food leads to increased depolarization of ASH relative to off food activity. Such changes in electrical activity are reflected behaviorally; organisms respond more robustly to copper if food is present [32].

*npr-9(GF)* organisms do not exhibit behavioral changes in response to food suggesting that the integration of food derived signals is inhibited (Figs 1, 2 and 3). A modified food race assay was utilized to evaluate if *npr-9(GF)* organisms can reach and maintain themselves on a bacterial food patch over the course of a 4 hour assay [43]. Moreover, a repulsive stimulus, i.e. copper, was also incorporated into the assay to evaluate if the abnormal aversive behavior of *npr-9(GF)* organisms would persist for extended periods of time. Coupling of attractant and antagonistic sensory cues has proven useful in characterizing the molecular underpinnings of behavioral choice [44]. Intuitively, the requirement for nutrition, i.e. food, is more important than exposure to potentially harmful environmental conditions if an organism is forced to choose between the two. As a consequence, the copper food race assay evaluates behavioral choice between an attractant (food) and an aversive cue (copper) over an extended period of time [44].

**Fig 9:**
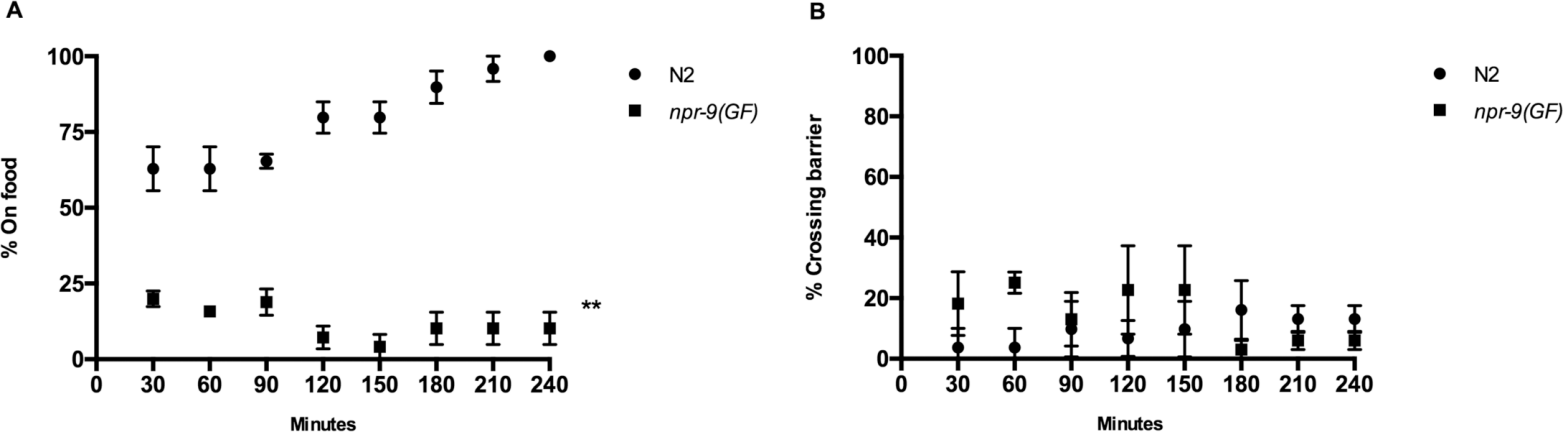
*npr-9(GF)* does not integrate food cues to modulate locomotory and aversive behavior to copper. (A) N2 and *npr-9(GF)* were evaluated for their ability to reach and stay on food during the copper-modified food race assay. (B) The aforementioned strains were evaluated for copper aversion in the absence of food in which positive responses are scored as crossing the copper barrier. Data are presented as mean +/- SE and at least 10 animals were assayed in three independent experiments analyzed with a two-way RM ANOVA. ** p< .01, significantly different from N2 animals under identical conditions.

Wildtype organisms are able to cross the copper barrier, find the food patch, and alter their locomotory behavior to maintain themselves on food (Fig 9A). Without food, nematodes rarely cross the barrier despite experiencing starvation conditions for 4 hours (Fig 9B). This indicates that nematodes can sense the presence of food across the copper barrier and that stimulation via food over-rides the aversive response as worms become more starved. We observed that *npr-9(GF)* animals would cross the barrier shortly after transfer to the plate and reach the food source. However, they did not maintain themselves on the food patch and would even re-cross the copper barrier rather than stay in a food-rich environment. Despite the ability to cross the copper barrier, *npr-9(GF)* organisms were incapable of modulating locomotory patterns to maintain themselves on the food patch (Fig 9A). Moreover, the absence of food did not alter aversive behaviors of *npr-9(GF)* mutants (Fig 9B). Provided that food should stimulate a higher aversive response, this suggests that upregulation of NPR-9 dependent signaling interferes with the integration of food cues [32].

## Discussion

Although the *C*. *elegans* nervous system is relatively simple, the organism is capable of detecting and integrating multiple environmental signals to modulate locomotion [4–7,9–12]. Consequently, *C*. *elegans* has proven to be a genetic model for the study of multisensory integration and sensorimotor control. Diversity in the distinct signaling pathways underlying nematode behaviors highlights that neural circuits can be regulated in an acute, i.e. sensory evoked, and tonic, i.e. spontaneous, fashion [3]. In mammalian models, an inability to integrate environmental cues has been linked to sensory processing disorders such as autism, epilepsy, and schizophrenia [1–2,45]. Although a number of neurons and signaling molecules have been identified, the characterization of the nematode locomotory circuit remains incomplete. AIB connectivity to a variety of sensory and inter- neurons indicates that the interneuron is extensively wired to integrate sensory evoked signals and regulate the interneuronal network [3,31].

### AIB coordinates interneurons of the spontaneous locomotory circuit

The pathway underlying spontaneous reversal frequency is regulated in a tonic fashion [17]. Acute inputs, reflective of novel environmental conditions, can modify AIB activity to shift from tonic to phasic regulation. The mechanisms that regulate such transitions in mammalian models have been impeded by the complexity of signaling pathways [46]. Analysis of AIB regulation in *C*. *elegans* has provided a more comprehensive view of the processes that mediate regulatory shifts [17].

Interneurons AVA and AIB function in parallel to initiate a spontaneous reversal on food; however, AVA regulation is primarily mediated via glutamate receptors, while AIB integrates serotonin, glutamate, and neuropeptide signals [9,11–13]. Activation of AVA or dis-inhibition of AIB can lead to a reversal [9]. In mammals, a similar sensorimotor circuit has been identified within the basal ganglia known as the ‘direct and indirect pathways model’ [47,48]. Initially, the direct and indirect pathways were believed to be distinctly regulated and have opposite effects on movement, however recent evidence has suggested that these circuits are structurally and functionally intertwined [48]. The presence of a synaptic connection from AIB to AVA suggests that these two circuits are structurally intertwined. Functional interactions between the AVA and AIB circuitries have also been identified in the regulation of locomotory responses to osmotic changes [9].

On food, AIB activity is inhibited via serotonin-gated chloride channel MOD-1 and dis-inhibition events trigger a reversal [17]. The presence of food and subsequent increased serotonin has no modulatory effect on the *npr-9(GF)* phenotypes indicating that excessive NPR-9 signaling interferes with the integration of serotonergic food cues. Similarly, serotonin pathways can reconfigure mammalian circuits and excessive galanin inhibits the integration of serotonin signals [49,50].

After a reversal event, nematodes recommence forward locomotion, alter their trajectory with minor turns, or perform an omega turn to facilitate a large directional change. Accordingly, the prevalence of omega turns is dictated by neuronal fine-tuning following reversal initiation. Fine-tuning of neurons in complex behaviors has also been highlighted in optic motor control and neuronal responses to opioids [51,52]. Such neuronal fine-tuning provides a mechanism that allows for a small population of neurons to mediate diverse and complex behaviors.

Recent evidence has highlighted that inhibitory and stimulatory glutamatergic inputs coordinate AIB activity to dictate behaviors following a reversal. However, up-regulation of NPR-9 can impede AIB dis-inhibition related to reversals and omega turns despite unique regulatory mechanisms underlying each behavior. Ablations to interneurons AIY, RIB, RIM, and RIV also alter omega turn frequencies indicating that the omega turn circuitry is likely regulated by outputs from a number of interneurons [24]. In agreement with a general inhibitory role, galanin broadly inhibits neuronal activity in mammalian models [53–55].

### Excessive galanin signaling prevents AIB integration of acute inputs

GLR-1 activity within either circuit, i.e. the activation of AVA or AIB, is sufficient for a nose-touch evoked reversal. The lack of reversals observed in *npr-9(GF)* animals suggests that signals from AIB can inhibit reversals initiated by AVA. Existing synaptic connections from AIB to AVA could mediate the transmission of modulatory signals [3]. Unlike spontaneous locomotion and octanol, the presence or absence of food does not modulate nose touch evoked backwards locomotion [10]. Moreover, the touch response is instantaneous, indicating that acutely stimulated pathways can operate on different timescales. Despite the different timescale and signaling modalities, excessive galanin receptor activation impedes multiple circuitries to inhibit backwards locomotion.

Analyses of on and off food octanol responses allows for the dissection of acute pathways that are reconfigured by the presence of food, i.e. context dependent circuit reconfiguration [56]. Similar circuit reconfiguration has also been shown to modulate vertebrate locomotory pathways [57]. Reversals stimulated via octanol are regulated by a complex signaling pathway that is coordinated by tyramine, octopamine, serotonin, glutamate, dopamine, and neuropeptides [15,17,58]. Octopamine is analagous to norepinephrine and its role in octanol signaling been likened to noradrenergic inhibition of nociception, i.e. pain, in mammals [59].

Inhibition of AIB, via a histamine-gated chloride channel, reduces spontaneous reversal frequency, yet produces a more rapid octanol response [17]. Despite the complexity of the octanol pathway, abnormal regulation of one neuron, i.e. AIB, via up-regulation of NPR-9, prevents the integration of any signal that could modify locomotory phenotypes. Similarly, up-regulation of galanin signaling in a mammalian context has been shown to impede the integration of food related environmental cues associated with Alzheimer’s disease and has been linked to antinociception in mammalian models [60, 61].

Responses to aversive stimuli facilitate the avoidance of unfavourable habitats. However, organisms exhibit less robust aversive responses the longer they are removed from food [6,62]. A reduction in serotonin signaling reconfigures neuronal networks to facilitate exploratory behavior to find a food source, while ignoring aversive cues [24]. Despite starvation conditions and altered neuronal regulation, upregulation of galanin signaling prevents locomotory modulation upon nematodes reaching a food source.

### The unique circuitry of the harsh touch response circumvents NPR-9 dependent inhibition

Interneurons AVA/AVD/AVE collectively regulate the initiation of some reversals, however each neuron has unique synaptic connectivity and receptors suggesting unique roles [3,11–13]. For example, AVD ablation does not differ from mock ablations relative to spontaneous reversals, but produces a reversal defect for harsh touch responses [9,36]. AIB is pre-synaptic to AVA and AVE, but is not pre-synaptic to AVD [3]. Provided that excessive galanin signaling does not inhibit harsh touch responses, the regulatory of influence of AIB is likely facilitated via synaptic transmission rather than extrasynaptic mechanisms.

### The lack of reversals impedes chemotaxis in support of the pirouette model of chemotaxis

*C*. *elegans* sense fluctuations in odorant concentration rather than absolute concentration, thus locomotory patterns must somehow accommodate changes in direction. Changes in reversal behavior (pirouette model) and/or changes in turning behavior (weathervane model) have been proposed to explain how modulation of locomotion can enable the detection of different attractant concentrations [19–21]. Conversely, nematodes that exhibit a severely defective turning behavior are not defective for chemotaxis suggesting that turning behavior is non-essential for attractant detection [22]. The lack of chemotaxis in *npr-9(GF)*, a mutant that turns but does not reverse, reinforces that only reversals are essential to diacetyl chemotaxis. Similar to the pirouette model, the biased random walk facilitates bacterial chemotaxis [63].

In our research we have shown that *npr-9*, a galanin-like GPCR, is a key regulator of reversal frequency in response to diverse stimuli. The analysis of multiple behaviors has highlighted the essential role of NPR-9 in regulating the interneuronal circuitry and has reinforced that AIB is a key hub for the integration of diverse sensory outputs. Moreover, regulation via NPR-9 can be circumvented in neurons that are not post-synaptic to AIB (e.g. AVD). Our study illustrates that excessive galanin signaling impedes the integration of environmental cues in AIB and that NPR-9 broadly coordinates the interneuronal circuitry despite expression limited to a single interneuron.

## Materials and Methods

### *C*. *elegans* culture

*C*. *elegans* were maintained at 20°C on NGM agar plates seeded with OP50 *E*. *coli* according to standard protocols.

### Strains

The following strains were used: wild-type strain N2, IC683 *npr-9(tm1652)*, IC836 *quIS20* [*npr-9*::*npr-9*; *sur-5*::*gfp; odr-1*::*rfp]*, KP4 *glr-1(n2461)*, MT6308 *eat-4(ky5)*, CX3410 *odr-10(ky225)*.

### Transgenic strains

Germline transformations were performed according to standard protocol. For *glr-1* KD, 50 ng of *odr-1*::RFP plasmid was used as the co-injection marker with 50 ng of the *glr-1* KD plasmid.

### Plasmid construction

#### *glr-1* KD

Andrew Fire vector pPD96.41 (empty backbone plasmid) was digested with *Hind*III and *Sma*I. A 2,002 bp *npr-9* promoter was PCR amplified from WT genomic DNA. The promoter was then cloned in the forward orientation with *Hind*III and *Sma*I and inserted into these sites. The *npr-9* promoter was also cloned in the reverse orientation using *Bgl*II and *Xho*I to generate a plasmid with a forward and reverse orientation *npr-9* promoter. A 371 base pair segment of the *glr-1* coding region was PCR amplified from the cDNA library and cloned into the vector with *Nhe*I and *Kpn*I digestion sites. The *glr-1* cDNA insert was cloned between the forward and reverse orientation promoters to produce double sided RNA of *glr-1* wherever *npr-9* is expressed (i.e. AIB). Primer sequences are included in S1 Table.

### Behavioral assays

For each assay L4 hermaphrodites were picked 16–24 hours prior to the start of an assay. All assays were performed at 20–23°C. Nematodes that were damaged during transfer or roamed off of the plate were not included in the data set. For on food reversal frequency assays, plates were seeded with 200μl OP 50 *E*. *coli* bacteria. The bacteria was then distributed to cover the entire plate. Seeded plates were incubated overnight and allowed to dry prior to the assay. Reversal frequency assays were performed as previously described [64]. Briefly, a young adult worm was transferred to a seeded an assay plate and allowed to acclimate for 1 minute prior to the start of the assay. Reversals were counted manually over a three minute period. Off food reversal frequency measurements were performed similarly; however, worms were transferred to an intermediate plate with no food for 1 minute prior to the assay start time to ensure that bacteria would not be carried over. A nematode was transferred from an intermediate plate to an assay plate with M9; excess M9 was removed delicately with a kim-wipe. Nose touch, octanol, and diacetyl assays were performed as previously described [6,65–67]. Nose touch assays were carried out in the absence of food. A hair was placed in front of a forward moving worm so that the organism collides with the hair at a perpendicular angle. Each organism was tested 10 times for nose touch and data was recorded as % responding. For octanol assays, responsiveness was determined by placing a hair dipped in 30% octanol in front of a forward moving organism. On food assay plates were prepared as previously mentioned. Off food octanol assays were performed after the nematode remained off food for ten minutes. Diacetyl assays were performed with a minimum of 50 worms, as dispersal timing can be population density dependent. Diacetyl assay plates were prepared as follows: 4 equal quadrants were marked on the underside of the plate with a 0.5 cm radius circle marked in the middle of the plate designated the “origin”. A point was marked in each quadrant that was equidistant from the origin and other quadrant points. Two points were designated as controls (EtOH and 0.5M sodium azide) or test (0.5% diacetyl diluted with EtOH and 0.5M sodium azide). 50–250 nematodes were washed in S Basal buffer and transferred to the origin with test or control solutions added to their designated points after transfer. The number of organisms outside the origin were counted to calculate the chemotaxis index. The “diacetyl tap assay” is a modified version of the previously described diacetyl assay; the modification involves the use of a plate tap to induce a reversal. Plate taps are performed once every 15 seconds throughout the one-hour diacetyl assay. The plate is rotated 90 degrees after each plate tap to prevent directional bias. The individual analysis of diacetyl responses to tap utilized a previously described chemotaxis assay, in which a radial gradient was established and a gradient peak is clearly marked on the plate [21]. Plate taps were incorporated into this assay and administered throughout. Single nematodes were tested for the ability to orient towards the attractant peak after tap application while moving away or towards the peak. A minimum of 5 trials per organism were used to evaluate tap responses. The copper-modified food race assay was designed based on a previously utilized food race assay and behavioral choice assay [43,44]. Plates were seeded with OP50 *E*. *coli* with a food patch that was positioned to one side of the plate rather than central. After the seeded plates were incubated overnight to allow for bacterial growth, approximately 100 μl of a 0.5M copper (II) sulfate solution was applied to an agar plate to create a barrier that divided the agar plate in two sections. This solution was also applied around the edge of the plate to prevent organisms from leaving the assay plate entirely. Once the copper (II) sulfate solution had dried, organisms were transferred to the section of the plate with no food with M9. Excess M9 was removed with a kim-wipe. Every 30 minutes worms were positively scored if they crossed the copper barrier and were observed on food. In the absence of food, positive responses were scored if nematodes crossed the copper barrier.

## Supporting Information

S1 Table Primer sequences for *glr-1* KD construct. (DOCX)
