# Supplementary material for: NPR-9, a Galanin-Like G-Protein Coupled Receptor, and GLR-1 Regulate Interneuronal Circuitry Underlying Multisensory Integration of Environmental Cues in Caenorhabditis elegans
Source: PLoS Genet. 2016 May 25;12(5):e1006050. doi: 10.1371/journal.pgen.1006050 (PMC4880332; doi:10.1371/journal.pgen.1006050)
Supplement: S1 Table — (DOCX) [file pgen.1006050.s001.docx]

**Table S1. Primer sequences for *glr-1* KD construct.**

Primer Primer Sequence

*npr-9* promoter For: 5’*-*CAGGCCAGAACTATTCGGGC*-*3’

*npr-9* promoter Rev: 5’*-*TTCCCAGGAAGTAGCTCTAA*-*3’

*glr-1* coding region For: 5*’-*GCCCTCCAAGAACGTGGTGAA*-*3’

*glr-1* coding region Rev: 5’- GGCAAGAATGCAGCTACCTG*-*3’
